# The Genus Heterogynis Rambur, 1866 (Heterogynidae, Lepidoptera): Congruence of Molecular, Morphological and Morphometric Evidence Reveal New Species in Serbia

**DOI:** 10.3390/insects14050455

**Published:** 2023-05-11

**Authors:** Dejan V. Stojanović, Vladislava Galović, Tomislav Terzin, Jelena Ačanski, Marija Vidović, Saša Orlović

**Affiliations:** 1 Institute of Lowland Forestry and Environment (ILFE), University of Novi Sad, Antona Čehova 13, 21000 Novi Sad, Serbia; dejanstojanovic021@yahoo.co.uk (D.V.S.); galovic@uns.ac.rs (V.G.); sasao@uns.ac.rs (S.O.); 2 Department of Science, Augustana Faculty, University of Alberta, 4901-46 Ave, Camrose, AB T4V 2R3, Canada; 3 BioSense Institute, University of Novi Sad, Dr Zorana Đinđića 1, 21000 Novi Sad, Serbia; acanski@biosense.rs; 4 Institute of Molecular Genetics and Genetic Engineering, University of Belgrade, Vojvode Stepe 444a, 11042 Belgrade, Serbia; mvidovic@imgge.bg.ac.rs

**Keywords:** DNA barcode, Heterogynidae, *Heterogynis serbica* sp. nov., morphometry, new taxon, scanning electron microscopy, Serbian *penella* group

## Abstract

**Simple Summary:**

The genus *Heterogynis*, with its wingless females and males with uniformly dull-coloured wings that show the same translucent grey–black colour, is certainly not the most exciting group of lepidopterans. However, this genus is interesting due to its relatively high divergence of DNA barcode. This high molecular divergence indicated that there might be an overlooked morphological diversity beyond the taxonomic characters usually analysed for species identification and delimitation. A new species, *Heterogynis serbica* sp. n. (Lepidoptera: Zygaenoidea, Heterogynidae) has been described from the Republic of Serbia, Balkan Peninsula, by applying an integrative taxonomic approach using morpho-anatomical characteristics, linear wing morphometry and DNA barcoding. Male genitalia, SEM analysis of adult male head anatomy, abdominal tergites/sternites, cocoons and habitats of closely related species *H. serbica* sp. n. and *H. zikici* are discussed and illustrated. Integrative taxonomy has shown its strength in species delimitation. Here, by integrating the results obtained using comparative SEM analysis, morphometry and COI-based DNA barcoding, it was determined that the individuals found on Mt. Kopaonik belong to a new species for science.

**Abstract:**

The Heterogynidae are a small family of moths consisting of a single genus *Heterogynis* and sixteen described species distributed in the Mediterranean region. A species new to science, *Heterogynis serbica* sp. nov., is described from the locality of Srebrenac, Mt. Kopaonik, Republic of Serbia, Balkan Peninsula, by applying an integrative taxonomic approach using morpho-anatomical characteristics, wing morphometics and DNA barcoding. Male genitalia, scanning electron micrographs of adult male head anatomy, abdominal tergites/sternites, cocoons and habitats of the closely related species *H. serbica* sp. nov. and *H. zikici* are discussed and illustrated. Photographs of adult males and females, cocoons, plants in which the cocoons were found and habitats are shown. Importantly, marked differences in genital structure and other morphological characters were noted. These differences were confirmed with forewing morphometrics and COI-based DNA barcoding results. Additionally, DNA barcodes for *H. serbica* sp. nov. and *H. zikici* were compared against previously available data for the genus to evaluate the phylogenetic relationships. We conclude that deep, previously unknown and unexpected intrageneric morphological diversity exists in the genus *Heterogynis*.

## 1. Introduction

Heterogynidae Rambur, 1866 is a small family of moths comprised of a single genus Heterogynis Rambur, 1866. This family is related to the Zygaenidae (Lepidoptera: Zigaenoidea, Heteroginidae). The males have translucent wings covered with filiform scales, and their proboscis and palps are reduced and bear pectinate antennae. The females are wingless and look like caterpillars [1]. The caterpillars are aposematically coloured. They weave a loosely covering cocoon on the stems of shorter plants. The males fly during the day.

There are sixteen described Heterogynidae species in the Mediterranean region, identified mainly on the basis of morphological and bionomic characters [1,2,3]. According to works by de Freina [3,4] clear differences in genital structure exist only between species complexes. A faunistic study on the species group *Heterogynis penella* (Hübner, 1818–1819) from the Balkans was presented by de Freina [5].

An integrative taxonomy has become the standard in the study of insect diversity. Due to the integration of multiple approaches, it is possible to reveal hidden diversity. Here, we integrated the results obtained using the analysis of morphological traits, scanning electron microscopy (SEM), wing morphometrics and DNA barcoding. The advantage of SEM is that it gives us more detailed insights into morphology and provides much new information that is not available with traditional morphological examination [6]. Wings are highly heritable structures and thus are suitable for morphometric studies in lepidopterans [7,8,9,10,11,12]. The majority of modern phylogenetic studies widely rely on the mitochondrial cytochrome c oxidase subunit I gene (COI) for species delimitation in animals [13]. A standardised region of the COI gene, named DNA barcode, has been an extensively used identification system for animal life [14]. Its success is already proven in the Heterogynidae taxonomy [5]. 

In this study, we compared new material collected in southwestern Serbia (Mt. Kopaonik) with other Serbian species from the *H. penella* species group—*Heterogynis zikici* [5]—collected 150 kilometres apart on the significant altitudinal gradient in south-eastern Serbia (Vlasina Lake). Here, we describe a new species based on SEM morpho-anatomical characteristics, morphometric analysis of the forewings and comparative genetic analysis of the COI gene (DNA barcode). In addition, we present the phylogenetic relationships in all available data for the genus *Heterogynis*. 

## 2. Material and Methods

### 2.1. Collection and Morphological Analysis

This study was conducted from May to August in a long-term interval from 2010 to 2020 at the site of Srebrenac, Mt. Kopaonik, Republic of Serbia, Balkan Peninsula. The moths were sampled in forest clearings, roads and subalpine and alpine meadows. Several standard methods were used to collect specimens and monitor moths. The cocoons were collected, packed, and brought to the laboratory for further analysis, determination and photographing. The adult insects were collected with the help of an entomological net and brought to the laboratory. Prof. Dr. Vladimir Žikić from the University of Niš, Faculty of Sciences and Mathematics, Department of Biology and Ecology, provided the analysed samples of *H. zikici*. All adults were subsequently preserved as dry specimens in entomological boxes. The holotype was deposited at the University of Novi Sad, the Institute of Lowland Forestry and Environment: Collection of Holotypes: D.V. Stojanović.

Permanent slides of the genital structure were deposited in the collection of permanent slides of D.V. Stojanović. For correct identification, the copulatory organs of adult males were dissected and then boiled in a water bath. Finally, the permanent microscope slides were made. The identification was conducted according to similar studies [1,5]. For the detailed taxon analysis, several literature sources were used [1,2,3,4,5,15,16,17,18,19,20,21,22,23].

The most prominent morphological features of the specimens were photographed with a Carl Zeiss Stemi 2000 stereomicroscope. The photos were processed using Motic^®^ images Plus 3.1 stacking software, and later, all images were formatted using Adobe Photoshop 2022 software, version 24. The photos of the site and wings for morphometric analysis were made using a Canon EOS 5D digital camera (Tokyo, Japan) equipped with a Canon lens EF 50 mm, 1:1.8 L. The photos of prepared specimens were taken in the laboratory of the Institute of Lowland Forestry and Environment, University of Novi Sad. In addition, photos of *H. serbica* sp. nov. and *H. zikici* male imagoes were taken using scanning electron microscopy (SEM) in high vacuum with secondary electron emission (SEI) at the Faculty of Natural Science, University of Novi Sad. For this purpose, the specimens were prepared in BAL-TEC SCD005 sputter coater for the work on JEOL JSM 6460 LV scanning microscope coupled with energy-dispersive X-ray spectroscopy (EDS; INCA, Oxford, UK).

### 2.2. Morphometric Analysis

Morphometric analysis of the forewing was conducted on a total of twelve male specimens: eight specimens of *H. zikici* and four specimens of *H. serbica* sp. nov. Six linear distances that could be reliably identified across the forewing were measured. The measurements were repeated three times, and an average of each linear distance was presented. The linear distances were labelled as a (wing length), b, c (wing widths), and d, e and f (wing areas in relation to the media veins). Six variables that correspond with ratios of the linear measurements were used in the analysis: a/f, b/c, b/f, c/f, d/f and e/f.

In a morphometric analysis of the forewings, measurements were made using TpsDig 2.05 software [24]. Differences among the obtained distance ratios between *H. zikici* and *H. serbica* sp. nov. were tested with an ANOVA. Principal component analysis was applied to the entire dataset to examine variations in forewing shape between these two species. Distance ratios were meaningfully correlated with the loading factor greater than ±0.7. A discriminant function analysis was conducted on a subset of independent principal components to test significance among species and address classification success. All statistical analyses were performed in Statistica ver. 13 (TIBCO Software Inc. 2018. Statistica data analysis software system).

### 2.3. DNA Isolation, Amplification and Sequencing

Total genomic DNA was extracted from larvae and leg tissue of *H. serbica* sp. nov. and *H. zikici* using the Dneasy Blood & Tissue Kit (Qiagen, Santa Clarita, CA, USA) according to the manufacturer’s protocol. 

For the COI barcoding fragment amplification, three primer pairs were used, which were designed for the amplification of the mitochondrial COI gene from diverse metazoan invertebrates described in the literature (Table 1). 

We used LepF1/LepR1, originally designed as “universal” DNA primers for phylogenetic analyses at the species and higher taxonomic levels [3,14,25,26]. For the amplification of the 658 bp mtDNA fragments, LCO1490/HCO2198 were used for species-level assignments as per Hebert et al. [14]. To amplify 632 bp of the 3′ region of COI, “Jerry”/“Pat” primers were used, similar to the DNA barcoding of Hymenoptera and Formicidae species [27,28]. Double-stranded DNA was amplified in a 25 µL volume reaction with 50 ng µL^−1^ of isolated DNA. Amplification of the fragments was carried out using the Eppendorf thermo cycler (Eppendorf, Germany) with the following amplification program: two cycles of 30 s at 94 °C, 30 s at 50 °C and 1 min at 72 °C and 20 cycles of 30 s at 94 °C, 30 s at 44 °C and 1 min at 72 °C and the final extension step for 7 min at 72 °C. 

**Table 1:** Primer sequences used for amplification of COI-based DNA barcodes.

Primers for COI Genes	Primer Sequences	Species	Reference
LepF1	5′-ATTCAACCAATCATAAAGATATTG-3′	*H. affinis*	[3]
LepR1	5′-TAAACTTCTGGATGTCCAAAAAATC-3′	*H. cynetis*	
LEP-F1	5′-ATTCAACCAATCATAAAGATAT-3′;	*Astraptes fulgerator*	[25]
LEP-R1	5′-TAAACTTCTGGATGTCCAAAAA-3′		
LEP-R2	5′-CTTATATTATTTATTCGTGGGAAAGC-3′		
LCO1490	5′-GGTCAACAAATCATAAAGATATTGG-3′	Diverse metazoan invertebrate	[29]
HCO2198	5′-TAAACTTCAGGGTGACCAAAAAATCA-3′		
LCO1490	5′-GGTCAACAAATCATAAAGATATTGG-3′	Taxon barcode	[14,29]
HCO2198	5′-TAAACTTCAGGGTGACCAAAAAATCA-3′		
CO1-exF	5′-ATCGCCTAAACTTCAGCCATT-3′	*Cucullia umbratica*	[30,31]
TL-N-3017	5′-CTTAAATCCATTGCACTAATCTGCCATA-3′		
“Jerry”	5′-CAACATTTATTTTGATTTTTTGG-3′	*Crematogaster fraxatrix*	[27,28]
“Pat”	5′-TCCAATGCACTAATCTGCCATATTA- 3′		

Obtained PCR products were purified using a DNA Clean & Concentrator-100 kit (Zymo Research, Irvine, CA, USA) according to the manufacturer’s instructions. To confirm DNA amplification and quality, PCR products were visualised on 2% agarose gel with ethidium bromide. All three primer pairs successfully amplified the expected length size of the COI fragment. Purified PCR products were quantified using a spectrophotometer Biospecnano (Shimadzu, Kyoto, Japan) and sequenced in both directions. 

All three primer pairs successfully amplified the expected length size of the COI fragment. The sequence was deposited in the National Centre for Biotechnology Information (NCBI) under the Gene Bank accession number MW286118 (Sequence ID: COI_LCO1490; genome: mitochondrial; locus: cytochrome c oxidase subunit 1) named *H. serbica*. DNA barcoding and molecular analyses were performed in the laboratory for molecular research at the Institute of Lowland Forestry and Environment, University of Novi Sad.

### 2.4. Nucleotide Sequence Analyses

The COI nucleotide sequences of *H. serbica* and all related species were found in two databases: the barcode of life data system (BOLD; https://www.boldsystems.org/index.php/databases, accessed on 20 January 2023) and NCBI; (https://www.ncbi.nlm.nih.gov, accessed on 20 January 2023). COI nucleotide sequence was further blasted on TBLASTX (https://blast.ncbi.nlm.nih.gov/Blast.cgi?PROGRAM=tblastx&PAGE_TYPE=BlastSearch&LINK_LOC=blasthome, accessed on 30 March 2023). The COI nucleotide sequences of *H. serbica* and other related species were aligned and compared using the pairwise sequence alignment tool Emboss Needle (https://www.ebi.ac.uk/Tools/psa/emboss_needle/, accessed on 20 January 2023; [32]).

The wider alignment of COI nucleotide sequences from *H. serbica* sp. nov. with 32 query nucleotide sequences most related to *H. penella* species across the world in the NCBI database was performed using MEGA 7.0 software [33]. The p-distance was calculated by comparing 100 COI sequences registered in BOLD GeneBank. The evolutionary history was inferred using the neighbour-joining statistical method with the bootstrapping method of 1000 replications [34]. The evolutionary distances were computed using the maximum composite likelihood method [35] including transitions and transversions substitutions including third codons. The pairwise distance for the marker gene—COI—was calculated using the Kimura 2 parameter model [36]. All ambiguous positions were removed for each sequence pair. In total, 687 positions were used in the final dataset. 

## 3. Results

### 3.1. Taxonomy

Family Heterogynidae Rambur, 1866

*Genus Heterogynis* Rambur, 1837

**Figure 1:**
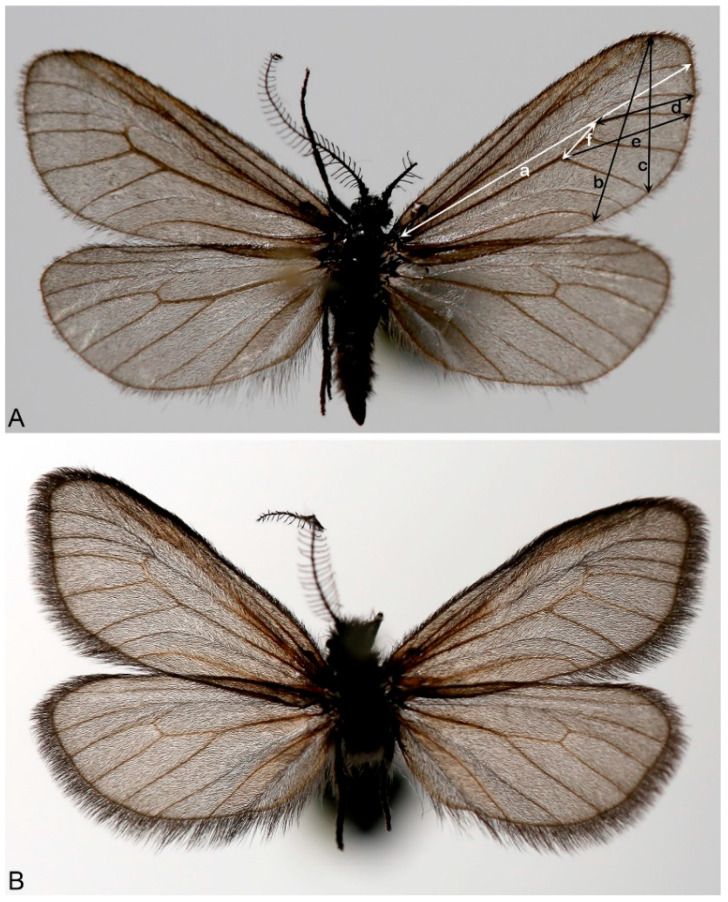
Illustration showing the wing measurements used in the morphometric analysis. (**A**) *H. zikici*, male. (**B**) *H. serbica* sp. nov., male. The linear distances were labelled as: a (wing length), b, c (wing widths), and d, e and f (wing areas in relation to the media veins). Six variables that correspond with ratios of the linear measurements were used in the analysis: a/f, b/c, b/f, c/f, d/f and e/f.

*Heterogynis serbica* Stojanović, Galović, Terzin, Ačanski, Vidović and Orlović sp. nov. (Figure 1).

Zoobank registration: urn:lsid:zoobank.org:act:0B44FDBA-2B4C-4738-BB45-7BA797CF7669.

*Deposited*. The University of Novi Sad, University of Novi Sad, ILFE: Holotype collection: D.V. Stojanović.

**Figure 2:**
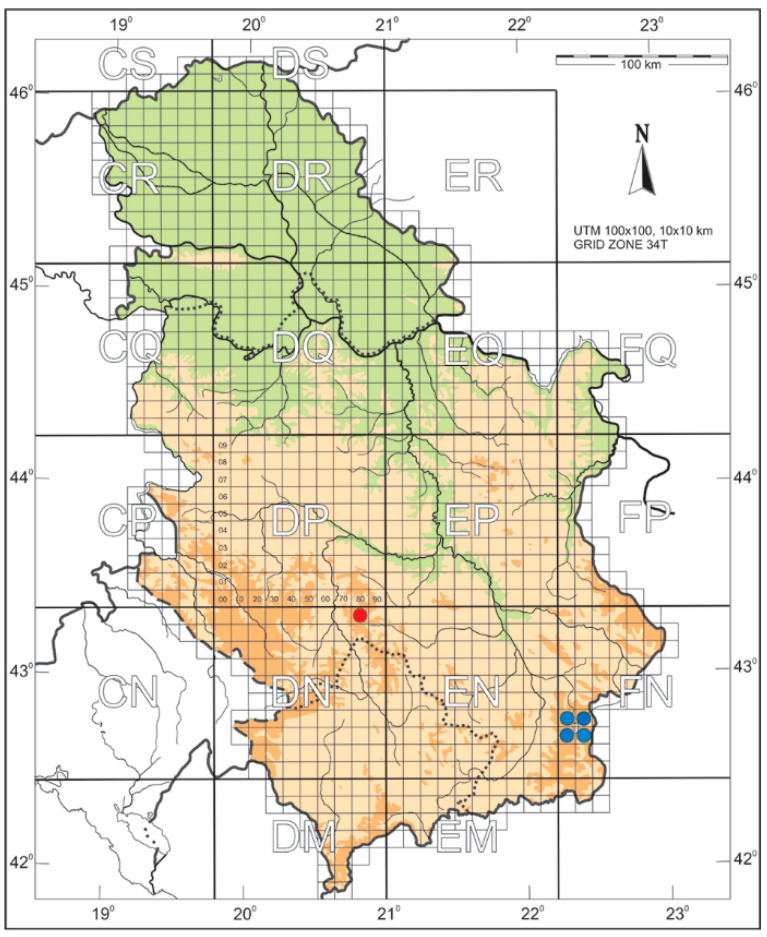
Map showing localities in Mt. Kopaonik and Vlasina Lake with UTM grids. Red point, *Heterogynis serbica* sp. nov.; blue points, *H. zikici.*

*Distribution*. Based on the summarized localities where the species were recorded or caught, a precise 10 × 10 km UTM map of the *H. serbica* sp. nov. (DN 89, red point) and *H. zikici* (FN 02, FN 03, FN 12, and FN 13, blue point) distribution in Serbia was made (Figure 2).

**Figure 3:**
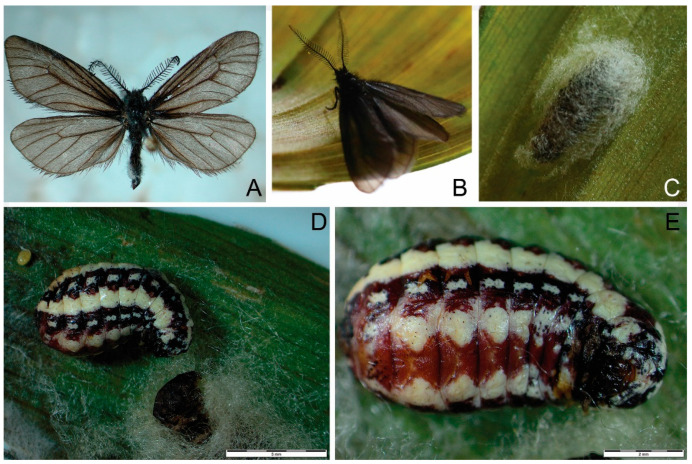
*Heterogynis serbica* sp. nov. type material. (**A**) Holotype, male, 17.4 cm. (**B**) Holotype, live habitus, male. (**C**) Paratype, female, cocoon. (**D**) Paratype, female, live habitus with cocoon. (**E**) Paratype, female, live habitus. Bar denotes 5 mm.

*Holotype.* Serbia, Srebrenac, about 80 km west of the city of Niš and about 30 km south of the city of Kraljevo, Serbian Alps, Mt. Kopaonik, 1600 m, 6 July 2020, D. V. Stojanović Institute of Lowland Forestry and Environment (ILFE) (Figure 3). A description of the holotype of *H. serbica* sp. nov. is given in Supplementary Figure S1.

*Description*. (*Heterogynis serbica* Stojanović, sp. nov.): ♂ (Figure 3A). Forewings: 9.1–9.6 mm (HT 9.2 mm), wingspan 17.4–19.1 mm (HT 17.4 mm), body length 6.1–6.7 mm (HT 6.7 mm).

*Paratype*. Serbia, Srebrenac, 5 mm, about 80 km west of the city of Niš and about 30 km south of the city of Kraljevo, Mt. Kopaonik, 1600 m, 18 June 2010, 17 July 2010, D. V. Stojanović (ILFE); 2 ff with cocoons, Mt. Kopaonik, Srebrenac 1600 m, 6 July 2020, D. V. Stojanović (ILFE); 2 ff Mt. Kopaonik, Srebrenac, on *Veratrum album* L. 1600 m, 28 June 2022, D. V. Stojanović (ILFE). The cocoon is silvery–white and scarce hairy (Figure 3C).

*Derivatio nominis*. We dedicate this species to the memory of Prof. Dr. Josif Pančić, the founder of Serbian Entomology, who called many taxa in his fruitful and long-term work “Serbica”.

### 3.2. Morphological Diagnosis

**Figure 4:**
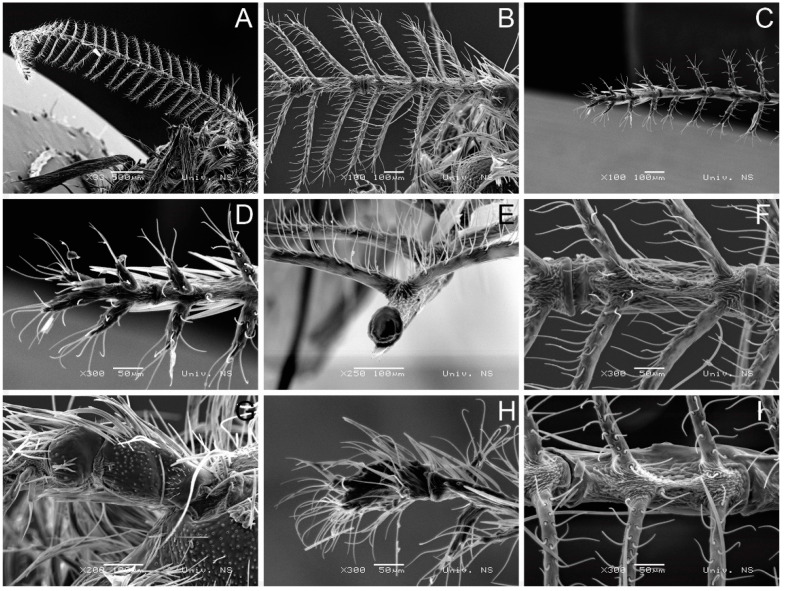
Scanning electron microscopy images showing micromorphology of bipectinate antennae of (**A**–**G**) *Heterogynis serbica* sp. nov. paratype. (**H**,**I**) *Heterogynis zikici.*

*H. serbica* sp. nov. is compared with *H. zikici*. The antennae of both species are shown in Figure 4. Leraut [1] described the antennae as pectinate, while De Freina [18] described the antennae as bipectinate. 

Upon detailed analysis of the data presented in Figure 4E, we concluded that *H. serbica* sp. nov. has a complex structure of antennae, where the pairs of branches are positioned under the angle of 240° lateral and 120° ventral with respect to the base of a segment.

The antennae of *H. serbica* sp. nov. are made of 15 segments (flagellum). Each segment carries two pairs of branches and contains a wide corridor between them. The pairs of branches are distant from the midline of a segment (Figure 4F). A similar structure of antennae has been observed in *H. zikici*, with two significant differences: first, the corridor between the pairs of branches on each segment is narrow; second, the pairs of branches are positioned closer to the midline of a segment (Figure 4I). 

The antennae of *H. serbica* sp. nov. contain the following parts: scape (base segment), which has a length of 282 μm, pedicellus, which has a length of 118 μm and flagellum made of 15 segments. In the direction from the pedicellus towards the tip of the antennae, the length of the first segment is 336 μm, the second segment has a length of 287 μm, the segment before the last segment is 191 μm and the last segment is 129 μm. Therefore, we can see a decrease in the length of antennal segments going from the base to the tip.

The tip of the antennae in *H. zikici* shows thickening (Figure 4H). This feature is absent in *H. serbica* sp. nov. (Figure 4C,D).

**Figure 5:**
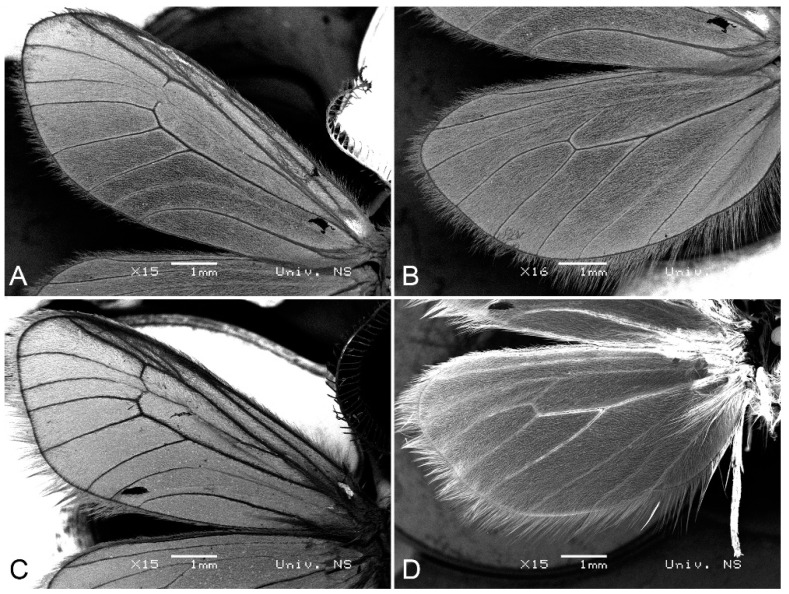
Scanning electron microscopy images. (**A**) Forewing of *Heterogynis zikici*. (**B**) Hindwing of *Heterogynis zikici*. (**C**) Forewing of *Heterogynis serbica* sp. nov. (**D**) Hindwing of *Heterogynis serbica* sp. nov. Bars denote 1 mm.

The wings of *Heterogynis serbica* sp. nov. and *H. zikici* are shown in Figure 5.

**Figure 6:**
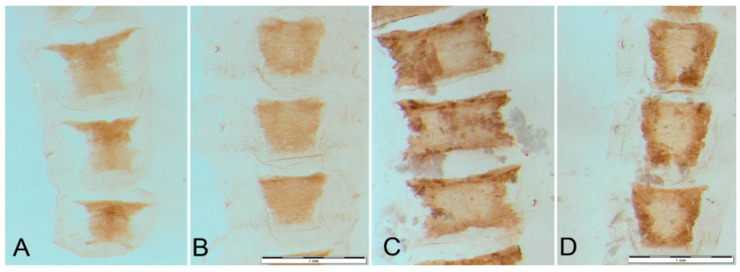
Abdominal plates of (**A**,**B**) *Heterogynis serbica* sp. nov. (**C**,**D**) *Heterogynis zikici*, sclerotization of tergites and sternites III–V. Bars denote 1 mm.

Abdominal segments of *H. serbica* sp. nov. are shown in Figure 6A,B. Sclerotization of tergites and sternites: *H. serbica* sp. nov. characteristic tergites III–V are cup-shaped, with narrowed bases and horned protrusions (Figure 6A); sternites are rounded (Figure 6B). Abdominal segments of *H. zikici* are shown in (Figure 6C,D). The tergites III–VII are wide and rectangular, with lateral serrated depression (Figure 6C). The sternites III–V are elongated (Figure 6D). In *H. serbica* sp. nov., the span of the horned extension of tergites is in the range of 795–1018 μm, while the base of the cup shape has a width of 492–509 μm (Figure 6A). In *H. zikici,* the respective measures are 1144–1172 μm at the top and 1058–1114 μm at the bottom (Figure 6C). The sternites in *H. serbica* sp. nov. are round, compared to the narrower sternites in *H. zikici*. 

**Figure 7:**
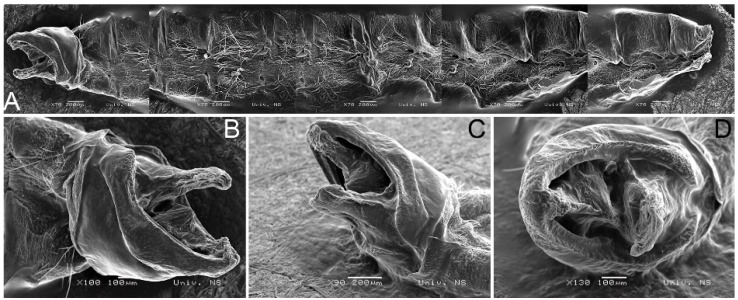
Scanning electron microscopy images showing *H. serbica* sp. nov. (**A**) Abdomen with male genitalia (lateral view). (**B**–**D**) Male genitalia.

The abdomen of *H. serbica* sp. nov. with male genitalia is shown in Figure 7.

**Figure 8:**
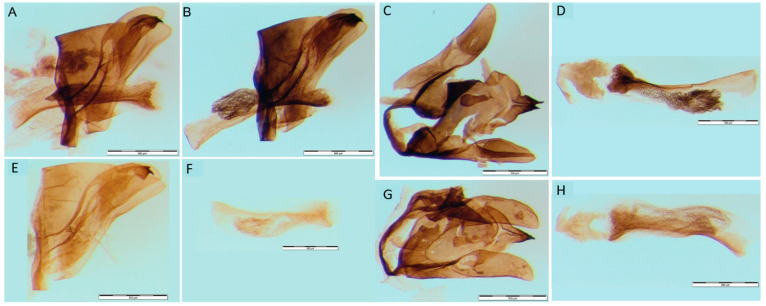
Male genitalia. (**A**) *Heterogynis serbica* sp. nov. profile view. (**B**) *Heterogynis zikici* profile view. (**C**) *Heterogynis zikici*. (**D**) *Heterogynis zikici* aedeagus. (**E**) *Heterogynis serbica* sp. nov. (**F**) *Heterogynis serbica* sp. nov. aedeagus. (**G**) *Heterogynis serbica* sp. nov. (**H**) *Heterogynis serbica* sp. nov. aedeagus. Bars denote 0.5 mm.

The male genital apparatus of *H. serbica* sp. nov. is significantly different in comparison to the male genitalia of *H. zikici* (Figure 8). The length of the aedeagus is different in the two species. The length of the aedeagus in *H. serbica* sp. nov. has a range of 1123–1221 μm (Figure 8A,F,H), while the length of the aedeagus in *H. zikici* has a range of 1074–1270 μm (Figure 8B,D). The width of the cucullus in *H. serbica* sp. nov. measured in the widest region is 216.67–296.58 μm (Figure 8A,E,G), which is significantly larger than the same structure in *H. zikici* at 197.00–221.79 μm (Figure 8B,C). The vesica in *H. zikici* is filled with small, clustered, dark spikes (Figure 8B,D), while the vesica in *H. serbica* sp. nov. contains numerous, small, scattered, pale spikes—cornuti (sclerotized plate) (Figure 8A,F,H). 

**Figure 9:**
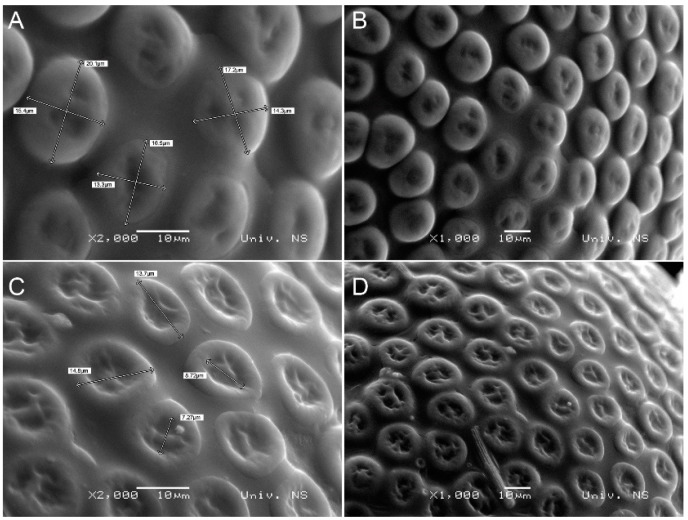
Scanning electron microscopy images showing the eyes of (**A**,**B**) *Heterogynis serbica* sp. nov. and (**C**,**D**) *Heterogynis zikici*.

The ocelli of the compound eye in *H. serbica* sp. nov. are prominently convex, with a diameter of 13.3–20.1 μm (Figure 9A,B). In *H. zikici*, the ocelli have a central depression and a diameter of 13.7–14.8 μm (Figure 9C,D). 

**Figure 10:**
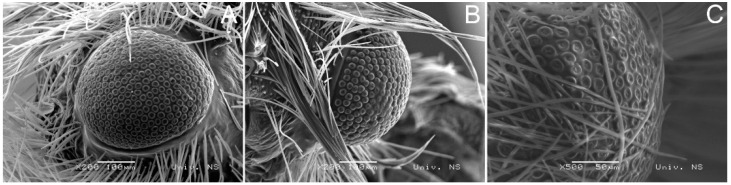
Scanning electron microscopy images showing the eyes of (**A,C**) *Heterogynis zikici* and (**B**) *Heterogynis serbica* sp. nov.

A frontal (Figure 10A) and lateral (Figure 10C) view of the compound eye of *H. zikici* and a lateral view of the compound eye of *H. serbica* sp. nov. (Figure 10B) are given.

**Figure 11:**
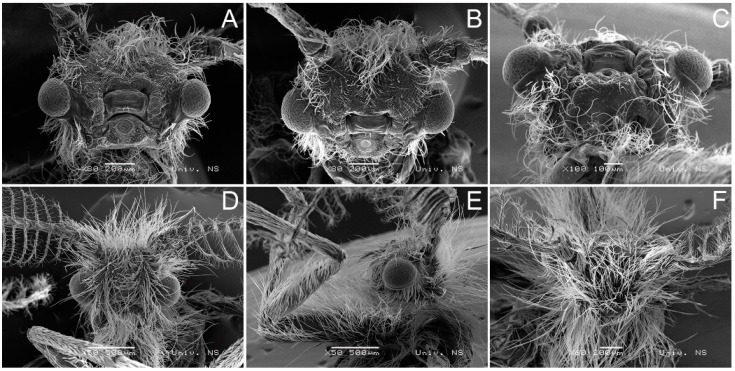
Scanning electron microscopy images showing the head of *H. zikici*. (**A**) The forehead, the mouth apparatus, the base of antennae and the compound eyes. (**B**) The forehead, the base of antennae and the compound eyes. (**C**) Head, ventral view. (**D**) Head, front view. (**E**) Head, lateral view. (**F**) Head, view from above.

Scanning electron microscopy images showing the head of *H*. *zikici* are shown in Figure 11. 

The eye morphology of Lepidoptera (Heterogynidae in particular) is an understudied area [37]. Although we believe that the presented results are valuable as the first demonstration of the intrageneric diversity in Lepidopteran compound eye morphology, the observed differences will not be further discussed.

**Figure 12:**
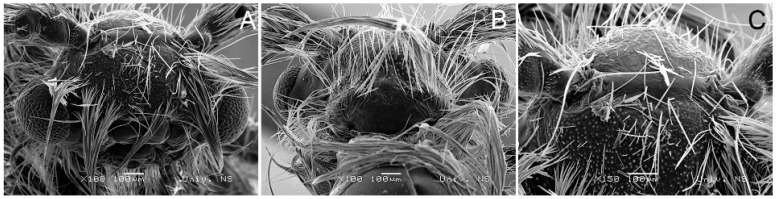
Scanning electron microscopy images showing the head of *H. serbica* sp. nov. paratype. (**A**) Frontal view. (**B**) Back of the head. (**C**) Inter-antennal protrusion or ridge.

A comparison of metric data for the head region of the two species according to SEM analysis (Figure 11 and Figure 12) is as follows:

In *H. serbica* sp. nov., the distance between the base of the antennae (scape) is 408 μm, which, at the same time, represents the width of the forehead ridge (Figure 12A,C). The distance between the compound eyes in *H. serbica* sp. nov. is 762 μm (Figure 12A). The diameter of the compound eyes in *H. serbica* sp. nov. is 350 μm (Figure 12A). The length of the clypeus measured at the longest part is 339 μm in *H. serbica* sp. nov. The width of the mouth opening is 93.7 μm (Figure 12A). The head region in males shows a strong reduction, with reduced proboscis and palpi (Figure 12A).

In *H. zikici*, the distance between the base of the antennae (scape) is 496 μm. The forehead ridge is absent (Figure 11A,B). The distance between the compound eyes in *H. zikici* is 716 μm (Figure 11A). The diameter of the compound eyes in *H. zikici* is 329 μm (Figure 11A). The length of the clypeus measured at the longest part in *H. zikici* is 320 μm. The width of the mouth opening is 50 μm (Figure 11A–C). The head region in males also shows a strong reduction, with reduced proboscis and palpi (Figure 11A–D), similar to *H. serbica* sp. nov. The head of *H. zikici* is hairier than *H. serbica* sp. nov. (Figure 11D–F). Most of the hairs were removed to take precise measurements of the *H. zikici* head (Figure 11A–C). In *H. serbica* sp. nov., hairs were not removed before taking the pictures (Figure 12).

**Figure 13:**
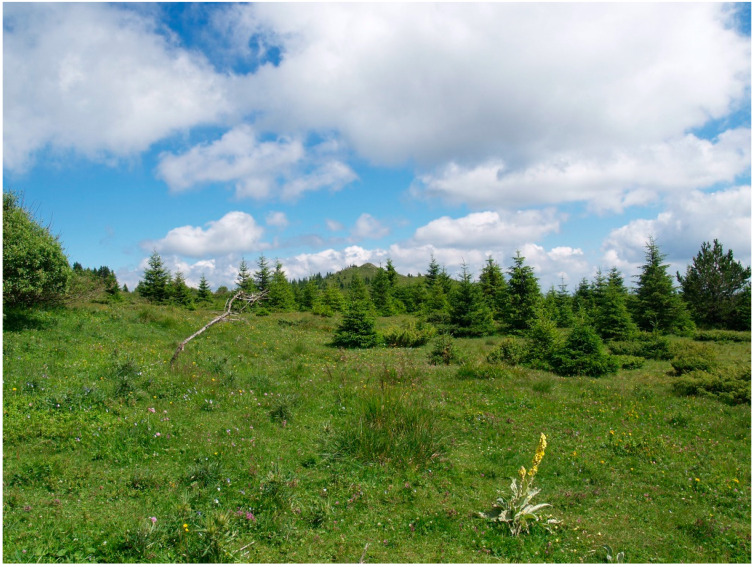
Habitat of *H. serbica* sp. nov. holotype, Mt. Kopaonik, Srebrenac, elevation 1600 m.

The habitat of *H. serbica* sp. nov. holotype Mt. Kopaonik, Serbian Alps, Srebrenac, 1600 m, shown in Figure 13, is positioned above the line of beech forest. It consists of sub-Alpine bushy clearings in the spruce forest zone. 

**Figure 14:**
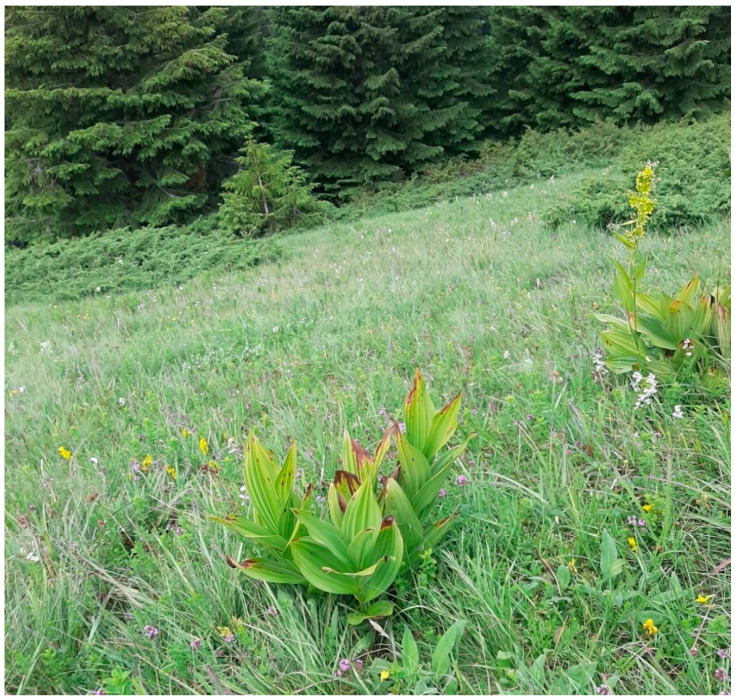
Type locality Mt. Kopaonik with plant species *Veratrum album* from which female specimens were collected.

The typical vegetation consists of dwarf juniper and wild blueberries scattered throughout a high mountain meadow. Females with cocoons were found on *Veratrum album* leaves (Figure 14).

The habitats of the two *Heterogynis* species are different, likely contributing to their speciation. The Ramsar Convention ranked the Vlasina as an area of extraordinary characteristics on the list of internationally significant wetlands. The habitat consists of meadows heavily interspersed with the host plant, which extends along the banks of the artificially created 16 km² Vlasina Lake, at an altitude of around 1200 m [38,39].

Grass communities of the *Nardetum* type are significant in the highest parts of Mount Kopaonik. They are found in the subalpine zone at altitudes between 1600 and 1900 m. These are most often thermophilic, sunny and dry habitats. The category of natural grasslands includes low-productivity grasslands that are often found on uneven terrains. This category also includes heaths and thickets [38]. The multiple differences in the two habitats mentioned above likely influenced the specialization in *Heterogynis* moths.

### 3.3. Morphometric Analysis

**Figure 15:**
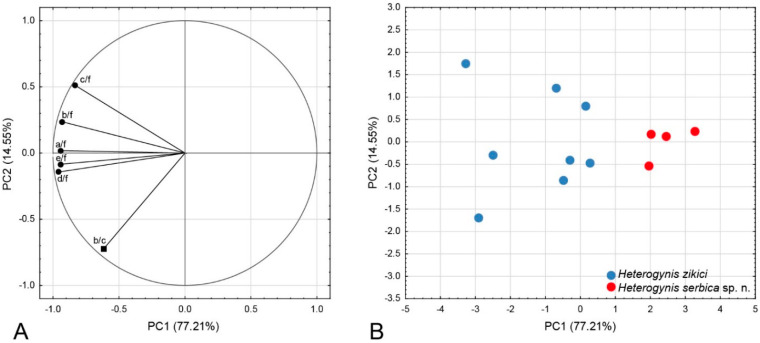
Forewing differences between *H. zikici* and *H. serbica* sp. nov. (**A**) Projection of the variables in the space defined by the PC1 and PC2 axes. ● variables correlated with PC1, ■ variable correlated with PC2. (**B**) Projection of the specimens in the space defined by the PC1 and PC2 axes.

The ANOVA revealed a significant difference between *H. zikici* and *H. serbica* sp. nov. in five out of the six analysed forewing distance ratios (a/f: F_1, 10_ = 14.112, *p* = 0.0037; b/c: F_1, 10_ = 4.83, *p* = 0.0527; b/f: F_1, 10_ = 21.686, *p* = 0.0009; c/f: F_1, 10_ = 11.5747, *p* = 0.0068; d/f: F_1, 10_ = 13.195, *p* = 0.0046; e/f: F_1, 10_ = 11.027, *p* = 0.0077). Moreover, the PCA conducted on the overall data set produced six PCs, from which the first two describe the majority of forewing variation (91.75%) (Table 2). Five ratios singled out as important for species delimitation with the ANOVA, a/f, b/f, c/f, d/f and e/f, were negatively correlated with PC1 (Figure 15A). This PC axis with the majority of the forewing variation (77.21%) clearly separated *H. zikici* and *H. serbica* sp. nov (Figure 15B). The PC2 axis was negatively correlated with the b/c ratio and depicts the population variability in *H. zikici* with 14.55% of forewing variation (Figure 15).

Additionally, the discriminant function analysis (DA) correctly classified all specimens to a priori defined groups based on the forewing shape variables. Additionally, the DA confirmed the separation of those two species based on forewing variables correlated with PC1 (Wilks’ Lambda = 0.96, *p* = 0.0037).

### 3.4. Molecular Analysis

**Figure 16:**
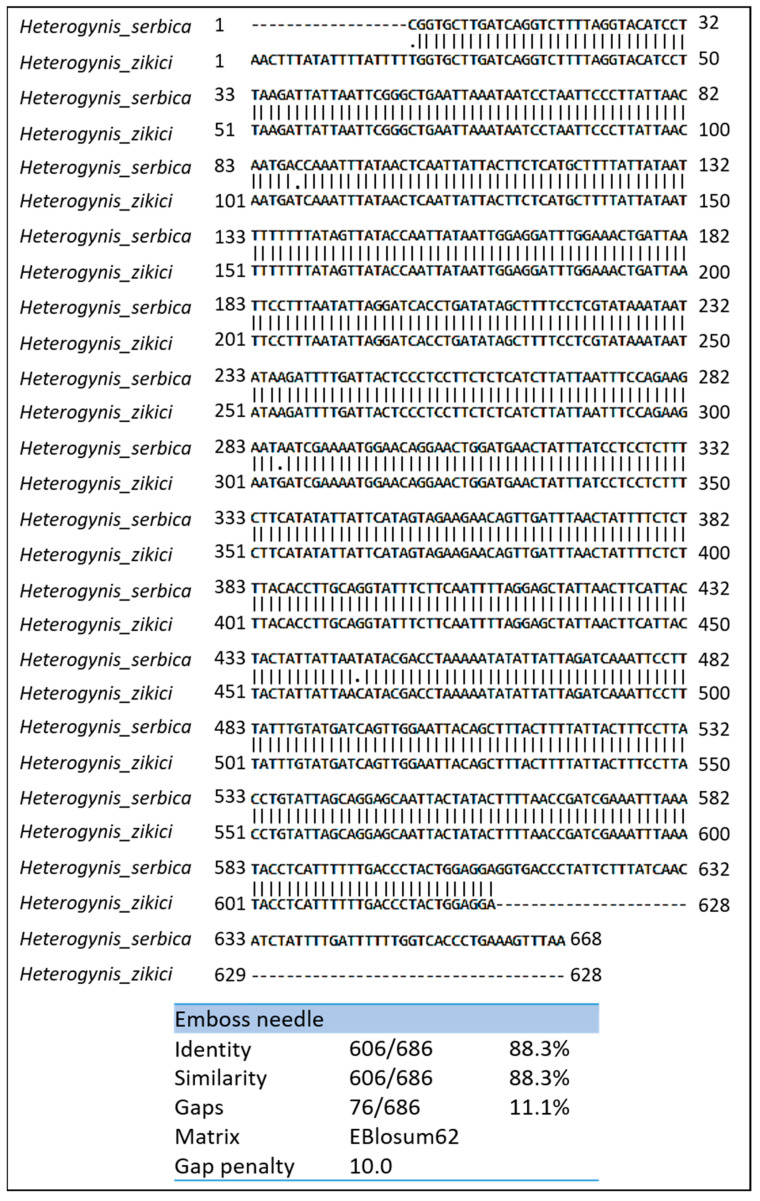
Nucleotide sequence variation in *H. serbica* and *H. zikici* COI genes.

The readable nucleotide sequence of the *H. serbica* sp. nov. COI barcode showed a fragment size of at least 668 bp. The nucleotide base composition (A = 204, T = 271; G = 79, C = 114) of the *H. serbica* sp. nov. mtDNA COI region showed, as in most other mitochondrial insect genes, poor G/C nucleotides (28%) while being rich in A/T nucleotides (71%). The alignment of the two sequences (*H. serbica* sp. nov. and *H. zikici*) showed 88.3% identities (606 bp of 686 bp; Figure 16). Moreover, the sequence alignment after removing the unaligned sites from the two ends showed 99.5 % identity (606/609, BLASTn, Supplementary Figure S2), revealing that three alterations in the partial nucleotide sequence of the *COI* gene were found. In addition, three point mutations, particularly transitions, were observed in, 87th base position downstream of the start codon of the *H. serbica* sp. nov. sequence (mutation C→T), 286th base position from the start codon (mutation A→G) and 446th base position in the sequence from the start codon (T→C).

**Figure 17:**
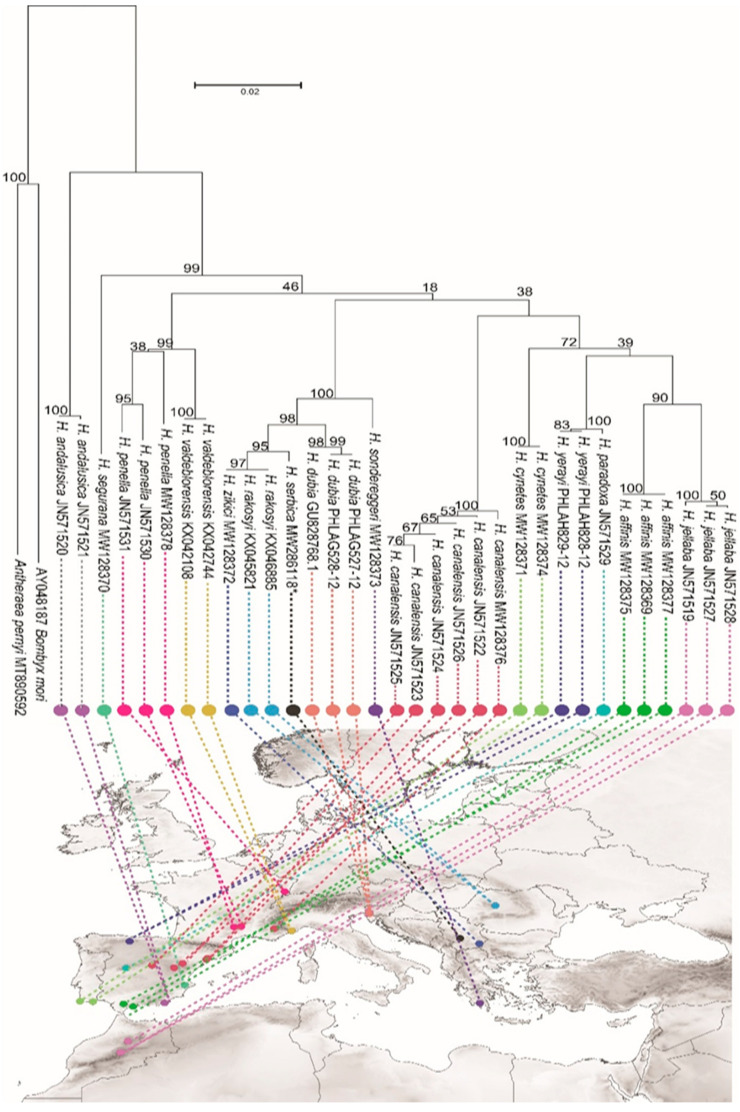
Neighbour-joining phylogenetic tree for the genus *Heterogynis* based on the COI mtDNA barcode plotted on a map of the Mediterranean basin. *Bombyx mori* and *Antheraea pernyi* were used as the outgroups.

In addition, we performed a multisequence alignment of the *H. serbica* sp. nov. COI gene sequence with those of 32 different *Heterogynis* sequences to evaluate the phylogenetic relationships between all specimens, particularly between *H. serbica* sp. nov. and *H. zikici*. The phylogenetic tree grouped all COI sequences into nine clades (Figure 17). 

The optimal tree with the sum of branch lengths of 0.68999121 is shown. The percentage of replicate trees in which the associated taxa were clustered using the bootstrap test (1000 replicates) is indicated next to the branches. The tree is drawn to scale, with branch lengths in the same units as the evolutionary distances in the heatmap of evolutionary, genetic divergence between sequences and variable sites (Supplementary Figure S3 and Table S1) used to infer the phylogenetic tree. The specimens within clades were closely related, and most of the clades were grouped by sequences belonging to the same species. The K2P value is a good indicator for the difference between the two species. The average K2P genetic distance between *H. serbica* sp. nov. and *H. zikici* was 0.7% (Supplementary Table S1) 

## 4. Discussion

The genus *Heterogynis*, with its wingless females and males with uniformly dull-coloured wings that show the same translucent grey–black colour throughout the taxon, is certainly not the most exciting group of lepidopterans when it comes to studying colour pattern diversity. However, a relatively high divergence in DNA barcodes, already known from the literature [3], indicated that there might be an overlooked morphological diversity beyond the taxonomic characters usually analysed for species identification and delimitation. Therefore, we decided to undertake a detailed morphological analysis of the newly collected specimens, described here as *H. serbica* sp. nov. and *H. zikici*. We described the new species, *Heterogynis serbica* sp. nov., using an integrative taxonomic approach that combines morpho-anatomical characteristics, wing morphometry and DNA barcoding.

For the first time, we present SEM images of the male heads and antennae of these two species, revealing deep morphological differences in the structure of compound eyes, antennae and overall head anatomy. Importantly, clear differences in the structure of the genital apparatus between these two species have been noted. To date, differences in this trait have been present only between species complexes [3,4]. In addition, detailed forewing morphometrics confirmed morphological differences between the two species. Using morphometrics, several authors have already shown that wing characteristics can serve as useful traits for delimitating Lepidoptera species [7,8,10,11]. Here, we confirmed the usefulness of morphometric analysis of forewings as important for species identification and classification.

COI barcoding is widely used in systematic studies of invertebrate metazoans. Upon DNA sequencing, the divergent existence of point mutations in the *COI* gene in *H. serbica* sp. nov. and *H. zikici* were determined. The COI-based DNA barcode analysis demonstrated that the interspecific divergence between *H. serbica* sp. nov. and *H. zikici* is within the expected values for the genus, clearly confirming that *H. serbica* sp. nov. is a new species within the known COI genetic margins for the genus Heterogynis. Therefore, although *H. serbica* sp. nov. and *H. zikici* share the same geographical origin, they are genetically different. Moreover, *H. serbica* sp. nov. is distant from all geographically neighbouring specimens based on mtDNA COI sequence regions. Although de Freina et al. [3] found no substantial mitochondrial COI sequence-based divergence in three specimens of *H. affinis* Rambur, 1837 that were 80 km apart on a straight line, here we found nucleotide mitochondrial COI sequence-based divergence between *H. serbica* sp. nov. and *H. zikici* on significant altitude difference (above 1600 m vs 1200 m). Apart from altitudinal differences, the habitats of these two species also differ in several characteristics.

The phylogenetic tree revealed that most clades were grouped by sequences belonging to the same species, mostly those derived from the same country. The sixth clade (100 bootstraps supported) consists of Slovenian and Italian COI sequences, and the seventh clade contains two Serbian COI sequences and two highly similar COI sequences from Romanian species. The comparisons showed that the COI sequences obtained from two species from Serbia were very close to the COI sequences of *H. rakosyi* [40] and *H. rakosyi* from Romania [41] (0.00–0.011) and belonged to the same clade even though *H. serbica* sp. nov. showed a higher genetic distance (0.011) and was distinguished from the *H. rakosyi* group. However, the COI sequence of *H. zikici* was identical to Romanian—*H. rakosyi* species (0.00), sharing the same group. The closest sixth clade to Romanian and Serbian (*H. serbica* sp. nov. and *H. zikici*) species consisted of COI sequences from *H. dubia* Schmidt, 1860 from Slovenia (3.312) and two identical from Italy (0.00), where all sequences share high homology. The Greek species *H. sondereggeri* [18] was connected to both clades from Serbia (*H. serbica* sp. nov., 0.060—*H. zikici* 0.069), Romania (0.069), and Italy (0.063). However, *H. sondereggeri* exhibited a significantly high genetic distance from Slovenian species (3.213) but connected to both distinct sixth and seventh Pennela groups. 

Considering that the distribution of the entire genus is related to the Mediterranean, the presence of genetically distinct species that are geographically closely related is not surprising. Furthermore, this newly discovered species is distributed in one of the three major European refugee areas, the Balkan Peninsula. The Balkan Peninsula has high biodiversity and endemism mainly due to a rich geological history. Many examples of insect species have altered their ranges and/or evolved as a response to repeated isolation during glacial–interglacial cycles across the Balkan Peninsula [42,43,44,45]. Similar studies on biodiversity in protected areas in Serbia indicate dynamic changes in species distribution due to climate change [46,47,48]. Some of these changes occur faster than we are able to record them, and some protected areas remain understudied and may harbour undiscovered species. Therefore, further expeditions across the Balkan Peninsula are necessary to investigate the presence of potential hidden diversity in this interesting group of moths.

## 5. Conclusions

Once again, integrative taxonomy has shown its strength in species delimitation. Here, by integrating the results obtained with comparative SEM analysis, morphometry and COI-based DNA barcoding, it was determined that the individuals found on Mt. Kopaonik belong to a new species for science. Although it could be expected that due to the geographical proximity, these individuals belong to the species *H. zikici*, our analyses undeniably show that it is a new species. Knowing the dynamics of the genus Heterogynis, it is unquestionable that there is a need for a comprehensive morphological and morphometric analysis of all the described taxa from this genus with more numerous samples of specimens to explore the extent of its diversity and possible adaptation meanings.

## Figures and Tables

**Table 2:** Eigenvalues from the Principal Component Analysis (PCA).

PCs	Eigenvalue	% Total (Variance)	Cumulative (%)
PC1	4.6324	77.21	77.21
PC2	0.87283	14.55	91.75
PC3	0.36108	6.02	97.77
PC4	0.11374	1.90	99.67
PC5	0.01984	0.33	99.99
PC6	0.00012	0.00	100.00

## Data Availability

The sequence of COI fragments of *Heterogynis serbica* Stojanović, Galović, Terzin, Ačanski, Vidović and Orlović sp. nov. were deposited in NCBI under the Gene Bank accession number MW286118 (Sequence ID: COI_LCO1490; genome: mitochondrial; locus: cytochrome c oxidase subunit 1) named *H. serbica*.

## Supplementary Materials

The following are available online at https://www.mdpi.com/article/10.3390/insects14050455/s1. Supplementary Figure S1. Description of the holotype *Heterogynis serbica* Stojanović sp. nov. Supplementary Figure S2. Nucleotide sequence alignment of *H. serbica* and *H. zikici* COI genes. Supplementary Figure S3. A heatmap showing the evolutionary divergence between sequences of 10 *Heterogynis* species. Supplementary Table S1. Pairwise K2P genetic distances between Cox1 sequences involved in the analyses.
