# Supplementary material for: The Genus Heterogynis Rambur, 1866 (Heterogynidae, Lepidoptera): Congruence of Molecular, Morphological and Morphometric Evidence Reveal New Species in Serbia
Source: Insects. 2023 May 11;14(5):455. doi: 10.3390/insects14050455 (PMC10231116; doi:10.3390/insects14050455)
Supplement: Supplementary file 1 [file insects-14-00455-s001.zip › Stojanovic et al updated supplements/Supplementary Figure S1_Description of the holotype.pdf]

Genus Heterogynis Rambur, 1837

Heterogynis serbica Stojanović, Galović, Terzin, Vidović, Ačanski & Orlović sp. n. (Figs. 1B, 3, 4A-G, 5CD, 6AB, 7, 8A,CD, 9CD, 10AB, 11B, 13)

Description of the holotype

Holotype. m (Fig. 3) SERBIA, Srebrenac, about 80 km west of the city of Niš and about 30 km south of the city of Kraljevo, Serbian Alps, Kopaonik mountain, 1600 m (Fig. 2), 6 July 2020, D. V. Stojanović (ILFE).

Wing-span of the front wings was 17.4 mm, and the length of the body (dry specimen) was measured from the tip of the head to the end of the abdomen at 6.7 mm (Fig. 3A).

Head: forehead contains a ridge positioned between antennae. Ocelli in compound eyes are prominently convex.

The segments of the antennae bipectinate; the length of the antennae is about two-thirds of the length of the front wings. Antennae are made of 15 segments with two pairs of branches, separated at angle of 120 degrees from each other. Antennal segments contain a wide corridor between the pairs of branches. The branches are positioned away from the segment midline. The tip of antennae (top bipectinate antennae) without thickening (Fig. 4C, D) is present in *H. zikici* (Fig. 4H).

Wings: color is uniform gray-black.

Abdominal segments with well-sclerotized parts of tergites III–VII, narrowed distally with horned protrusions; sternites III–VII rounded, narrowed distally.

Male genitalia: Tegumen laterally angled on the top, nearly flat (8A), valvae in the widest region thickened, convex (Figs. 8A, C, 9C).

Aedeagus bent, vesicafilled with numerous, pale, scattered, small spikes (scobinate) (Figs.8D, 9D).

Cocoon, hairy, lightly woven (Fig. 3C).
