# Supplementary material for: The Genus Heterogynis Rambur, 1866 (Heterogynidae, Lepidoptera): Congruence of Molecular, Morphological and Morphometric Evidence Reveal New Species in Serbia
Source: Insects. 2023 May 11;14(5):455. doi: 10.3390/insects14050455 (PMC10231116; doi:10.3390/insects14050455)
Supplement: Supplementary file 1 [file insects-14-00455-s001.zip › Stojanovic et al updated supplements/Supplementary Figure S2-1.pdf]

```
Heterogynis_zikici     2    GGTGCTTGATCAGGTCTTTTAGGTACATCCTTAAGATTATTAATTCGGGCTGAATTAAAT  61
                            ||||||||||||||||||||||||||||||||||||||||||||||||||||||||||||
Heterogynis_serbica    20   GGTGCTTGATCAGGTCTTTTAGGTACATCCTTAAGATTATTAATTCGGGCTGAATTAAAT  79

Heterogynis_zikici     62   AATCCTAATTCCCTTATTAACAATGACCAAATTTATAACTCAATTATTACTTCTCATGCT  121
                            |||||||||||||||||||||||||| |||||||||||||||||||||||||||||||||
Heterogynis_serbica    80   AATCCTAATTCCCTTATTAACAATGATCAAATTTATAACTCAATTATTACTTCTCATGCT  139

Heterogynis_zikici     122  TTTATTATAAttttttttATAGTTATACCAATTATAATTGGAGGATTTGGAAACTGATTA  181
                            ||||||||||||||||||||||||||||||||||||||||||||||||||||||||||||
Heterogynis_serbica    140  TTTATTATAATTTTTTTTATAGTTATACCAATTATAATTGGAGGATTTGGAAACTGATTA  199

Heterogynis_zikici     182  ATTCCTTTAATATTAGGATCACCTGATATAGCTTTTCCTCGTATAAATAATATAAGATTT  241
                            ||||||||||||||||||||||||||||||||||||||||||||||||||||||||||||
Heterogynis_serbica    200  ATTCCTTTAATATTAGGATCACCTGATATAGCTTTTCCTCGTATAAATAATATAAGATTT  259

Heterogynis_zikici     242  TGATTACTCCCTCCTTCTCTCATCTTATTAATTTCCAGAAGAATAATCGAAAATGGAACA  301
                            |||||||||||||||||||||||||||||||||||||||||||| |||||||||||||||
Heterogynis_serbica    260  TGATTACTCCCTCCTTCTCTCATCTTATTAATTTCCAGAAGAATGATCGAAAATGGAACA  319

Heterogynis_zikici     302  GGAACTGGATGAACTATTTATCCTCCTCTTTCTTCATATATTATTCATAGTAGAAGAACA  361
                            ||||||||||||||||||||||||||||||||||||||||||||||||||||||||||||
Heterogynis_serbica    320  GGAACTGGATGAACTATTTATCCTCCTCTTTCTTCATATATTATTCATAGTAGAAGAACA  379

Heterogynis_zikici     362  GTTGATTTAACTATTTTCTCTTTACACCTTGCAGGTATTTCTTCAATTTTAGGAGCTATT  421
                            ||||||||||||||||||||||||||||||||||||||||||||||||||||||||||||
Heterogynis_serbica    380  GTTGATTTAACTATTTTCTCTTTACACCTTGCAGGTATTTCTTCAATTTTAGGAGCTATT  439

Heterogynis_zikici     422  AACTTCATTACTACTATTATTAATATACGACCTAAAAATATATTATTAGATCAAATTCCT  481
                            ||||||||||||||||||||||| ||||||||||||||||||||||||||||||||||||
Heterogynis_serbica    440  AACTTCATTACTACTATTATTAACATACGACCTAAAAATATATTATTAGATCAAATTCCT  499

Heterogynis_zikici     482  TTATTTGTATGATCAGTTGGAATTACAGCTTTACTTTATTACTTTCCTTACCTGTATTA  541
                            |||||||||||||||||||||||||||||||||||||||||||||||||||||||||||
Heterogynis_serbica    500  TTATTTGTATGATCAGTTGGAATTACAGCTTTACTTTATTACTTTCCTTACCTGTATTA  559

Heterogynis_zikici     542  GCAGGAGCAATTACTATACTTTTAACCGATCGAAATTTAAATACCTCATTTTTTGACCCT  601
                            ||||||||||||||||||||||||||||||||||||||||||||||||||||||||||||
Heterogynis_serbica    560  GCAGGAGCAATTACTATACTTTTAACCGATCGAAATTTAAATACCTCATTTTTTGACCCT  619

Heterogynis_zikici     602  ACTGGAGGA  610
                            |||||||||
Heterogynis_serbica    620  ACTGGAGGA  628
```

| BLAST Seq alignment | | |
|---|---|---|
| Identity | 606/609 | 99.5% |
| Query cover | 91% | |
| Gaps | 0/609 | 0% |
