# Supplementary material for: The Genus Heterogynis Rambur, 1866 (Heterogynidae, Lepidoptera): Congruence of Molecular, Morphological and Morphometric Evidence Reveal New Species in Serbia
Source: Insects. 2023 May 11;14(5):455. doi: 10.3390/insects14050455 (PMC10231116; doi:10.3390/insects14050455)
Supplement: Supplementary file 1 [file insects-14-00455-s001.zip › Stojanovic et al updated supplements/Supplementary Figure S3.pdf]

**identity**	***H. serbica***	*H. zikici*	*H. penella*	*H. sondereggeri*	*H. canalensis*	*H. segurana*	*H. affinis*	*H. yerayi*	*H. rakosy*	*H. dubia*
H. serbica	**100**	**79.6**	**86.4**	**84.6**	**85.5**	**85.5**	**85.5**	**91.7**	**85.5**	**90.8**
H. zikici		**100**	80.2	78.7	79.4	79.4	79.4	80.9	82.6	82.6
H. penella			**100**	92.8	94.7	94.7	94.7	92.2	94.9	94.9
H. sondereggeri				**100**	95.1	95.1	95.1	89.5	93	93
H. canalensis					**100**	100	100	90.4	93.9	93.9
H. segurana						**100**	100	90.4	93.9	93.9
H. affinis							**100**	90.4	93.9	93.9
H. yerayi								**100**	95.4	95.4
H. rakosy									**100**	94.9
H. dubia										**100**

Correlation of COI sequence identity among 10 *Heterogynis* species according to Emboss needle software [8].

**similarity**	***H. serbica***	*H. zikici*	*H. penella*	*H. sondereggeri*	*H. canalensis*	*H. segurana*	*H. affinis*	*H. yerayi*	*H. rakosy*	*H. dubia*
H. serbica	**100**	**80.4**	**87.7**	**85.1**	**86**	**86**	**86**	**92.5**	**86**	**90.8**
H. zikici		100	81.4	79.1	79.8	79.8	79.8	82.1	82.6	82.6
H. penella			100	94.3	96.2	96.2	96.2	93.6	96.3	96.3
H. sondereggeri				100	96.1	96.1	96.1	90.9	93.5	93.5
H. canalensis					100	99.5	99.5	91.8	94.4	94.4
H. segurana						100	99.5	91.8	94.4	94.4
H. affinis							100	91.8	94.4	94.4
H. yerayi								100	96.3	96.3
H. rakosy									100	96.3
H. dubia										100

Correlation of COI sequence similarity among 10 *Heterogynis* species according to Emboss needle software [8].

COI Sequences among 10 *Heterogynis* species:

>BDE649-20_Heterogynis penella_France (penella)
TLYFIFGAWSGLLGMSLSLLIRAELNNPNSLINNDQIYNSIITSHAFIMIFFMVMPIMIGGFGNWLIPLMLGSPDMAFPRMNNMSFL
LPPSIILLISSSMIENGTGTGWTIYPPLSSYFIHSSSTIDLTIFSLHLAGISSILGAINFITTIINMRPKNMSLDQIPLFVWSVGITALLLLLSLP
VLAGAITMLLTDRNLNTSFFDPSGG

>BDE650-20_Heterogynis sondereggeri_Greece (penella)
TLYFIFGAWSGLLGTSLSLLIRAELNNPNSLINNDQIYNSIITSHAFIMIFFMVMPIMIGGFGNWLIPLMLGSPDMAFPRMNNMSFW
MLPPSLILLISSSMIENGTGTGWTIYPPLSSYIIHSSSTVDLTIFSLHLAGISSILGAINFITTIINMRPKNMLLDQIPLFVWSVGITALLLLLS
LPVLAGAITMLLTDWNLNT

>BDE651-20_Heterogynis canalensis_France (canelensis)

TLYFIFGAWSGLLGTSLSLLIRAELNNPNSLINNDQIYNSIITSHAFIMIFFMVMPIMIGGFGNWLIPLMLGSPDMAFPRMNNMSFW
LLPPSLILLISSSMIENGTGTGWTIYPPLSSYIIHSSSTVDLTIFSLHLAGISSILGAINFITTIINMRPKNMSPNQIPLFVWSVGITALLLLLSL
PVLAGAITMLLTDRNLNTLFFDX

>BDE652-20_Heterogynis segurana_Spain (penella)
TLYFIFGAWSGLLGTSLSLLIRAELNNPNSLINNDQIYNSIITSHAFIMIFFMVMPIMIGGFGNWLIPLMLGSPDMAFPRMNNMSFW
LLPPSLILLISSSMIENGTGTGWTIYPPLSSYIIHSSSTVDLTIFSLHLAGISSILGAINFITTIINMRPKNMSPNQIPLFVWSVGITALLLLLSL
PVLAGAITMLLTDRNLNTLFFDX

>BDE656-20_Heterogynis affinis_Spain (affinis)
TLYFIFGAWSGLLGTSLSLLIRAELNNPNSLINNDQIYNSIITSHAFIMIFFMVMPIMIGGFGNWLIPLMLGSPDMAFPRMNNMSFW
LLPPSLILLISSSMIENGTGTGWTIYPPLSSYIIHSSSTVDLTIFSLHLAGISSILGAINFITTIINMRPKNMSPNQIPLFVWSVGITALLLLLSL
PVLAGAITMLLTDRNLNTLFFDX

>PHLAH828-12_Heterogynis yerayi_Spain (paradoxa)
TLYFIFGAWSGLLGTSLSLLIRTELNNPNSIINNDQIYNSIITSHAFIMIFFMVMPIMIGGFGNWLIPLMLGSPDMAFPRMNNMSFWL
LPPSLILLISSSMIENGTGTGWTIYPPLSSYIIHSSSTIDLTIFSLHLAGISSILGAINFITTIINMRPKNMLLDQIPLFVWSVGITALLLLLSLPV
LAGAITMLLTDRNLNTSFFDPAGGGDPILYQHLF

>PHLAH047-12_Heterogynis rakosy_Romania (penella)
TLYFIFGAWSGLLGTSLSLLIRAELNNPNSLINNDQIYNSIITSHAFIMIFFMVMPIMIGGFGNWLIPLMLGSPDMAFPRMNNMSFW
LLPPSLILLISSSMIENGTGTGWTIYPPLSSYIIHSSSTVDLTIFSLHLAGISSILGAINFITTIINMRPKNMLLDQIPLFVWSVGITALLLLLSL
PVLAGAITMLLTDRNLNTSFFDPTGGGDPIX

>LEFIA1167_10_Heterogynis dubia_Slovenia (penella)
TLYFIFGAWSGLLGTSLSLLIRAELNNPNSLINNDQIYNSIITSHAFIMIFFMVMPIMIGGFGNWLIPLMLGSPDMAFPRMNNMSFW
LLPPSLILLISSSMIENGTGTGWTIYPPLSSYIIHSSSTVDLTIFSLHLAGISSILGAINFITTIINMRPKNMLLDQIPLFVWSVGITALLLLLSL
PVLAGAITMLLTDRNLNTSFFDPTGGGDPIX

>Heterogynis_serbica (internal ID 1CCDZAG273, GenBank ID_MW286118; Serbia)
GAWSGLLGTSLSLLIRAELNNPNSLINNDQIYNSIITSHAFIMIFFMVMPIMIGGFGNWLIPLMLGSPDMAFPRMNNMSFWLLPPSL
ILLISSSMIENGTGTGWTIYPPLSSYIIHSSSTVDLTIFSLHLAGISSILGAINFITTIINMRPKNMLLDQIPLFVWSVGITALLLLLSLPVLAG
AITMLLTDRNLNTSFFDPTGGGDPILYQHLFWFFGHPESL

>Heterogynis_zikici (BDE648-20, GenbankID, MW128372, Serbia)
TLYFIFGAWSGLLGTSLSLLIRAELNNPNSLINNDQIYNSIITSHAFIMIFFMVMPIMIGGFGNWLIPLMLGSPDMAFPRMNNMSFW
LLPPSLILLISSSMIENGTGTGWTIYPPLSSYIIHSSSTVDLTIFSLHLAGISSILGAINFITTIINMRPKNMLLDQIPLFVWSVGITALLLLLSL
PVLAGAITMLLTDRNLNTSFFDPTGGTLYFIFGAWSGLLGTSLSLLIRAELNNPNSLINNDQIYNSIITS
