# Supplementary figures and images for: The Genus Heterogynis Rambur, 1866 (Heterogynidae, Lepidoptera): Congruence of Molecular, Morphological and Morphometric Evidence Reveal New Species in Serbia
Source: Insects. 2023 May 11;14(5):455. doi: 10.3390/insects14050455 (PMC10231116; doi:10.3390/insects14050455)

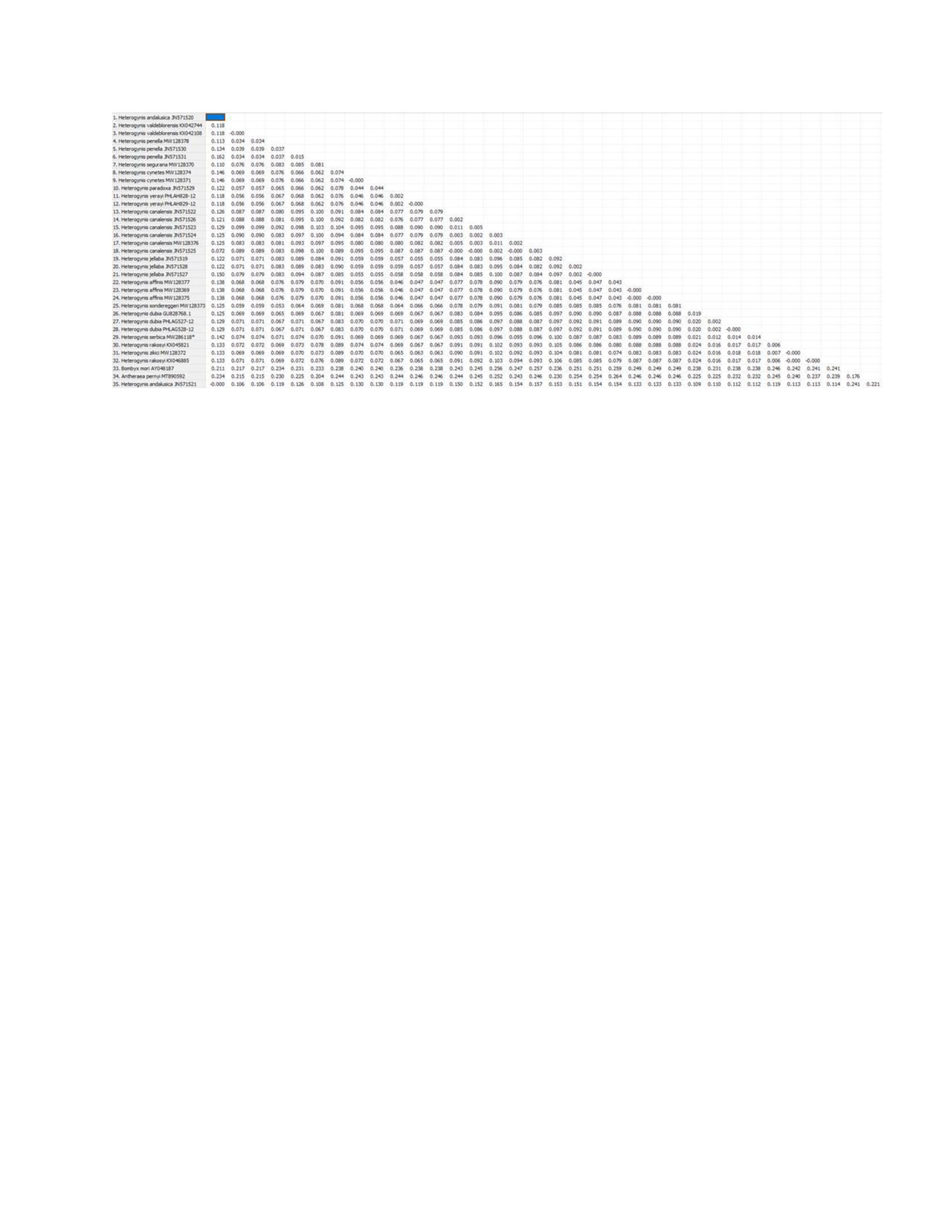

Supplement: Supplementary file 1 [file insects-14-00455-s001.zip › Stojanovic et al updated supplements/Supplementary Table S1-Pairwise K2P.jpg]
